# P-1447. Does Race or Social Vulnerability Play a Role in Antimicrobial Utilization for Admitted Patients? A Retrospective Analysis in a Large Healthcare System

**DOI:** 10.1093/ofid/ofae631.1619

**Published:** 2025-01-29

**Authors:** Reese Cosimi, Maria Mupanomunda, Stacy D Garrett-Ray, Florian Daragjati, Moyo Kimathi, frederick masoudi, Thomas Aloia, Richard Fogel, Mohamad G Fakih, Allison Bollinger

**Affiliations:** Ascension, Fishers, Indiana; Ascension, Fishers, Indiana; Ascension, Fishers, Indiana; Ascension, Fishers, Indiana; Ascension, Fishers, Indiana; Ascension/ Chief Science Officer, VP of Research and Anlaytics, St. Louis, Missouri; Ascension, Fishers, Indiana; Ascension, Fishers, Indiana; Ascension, Fishers, Indiana; Ascension, Fishers, Indiana

## Abstract

**Background:**

Rising antibiotic resistance is a major public health threat. In 2015 our organization responded by establishing antimicrobial stewardship programs in all acute care facilities. A review of the literature has shown concerning disparities in antibiotic use across different populations, potentially contributing to unequal health outcomes. Our organization has an expansive geographic footprint that encompasses diverse patient populations which precipitates the need to evaluate care across the system.

**Table 1.**
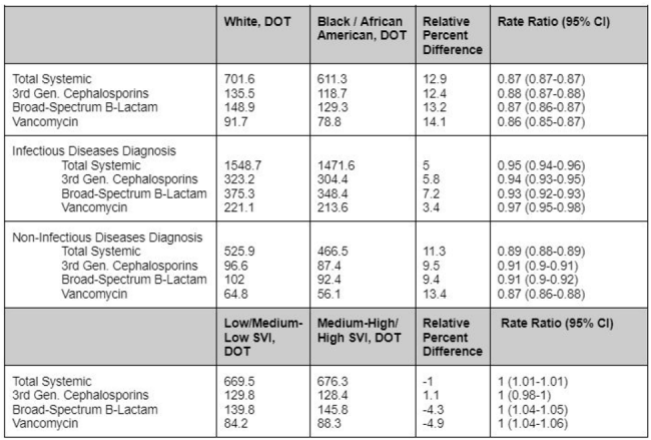
Antimicrobial Utilization by Race and SVI DOT - Days of Therapy per 1000 Days Present

**Methods:**

Retrospective analysis of 103 hospitals across a large healthcare system of all admitted patients who received a target antibiotic between January 1, 2023 to December 31, 2023. The primary clinical outcome was antimicrobial utilization defined as duration of therapy (DOT) per 1000 days present for four antibiotic groups (total systemic, 3rd generation cephalosporins, broad-spectrum beta-lactams, vancomycin). Data were stratified across patient race and social vulnerability index (SVI) to identify factors that may be contributing to gaps in equitable care.

**Results:**

A total of 665,312 patients were included in the analysis (70.5% White, 17.6% Black/African American (AA)). A greater proportion of White patients were 65 years and older compared with the Black/AA population which were primarily 18 to 64 years. Infectious disease diagnoses accounted for 14.6% of all encounters. Antibiotic utilization for White patients was 13% higher than Black/AA in Total Systemic antibiotics (*p*< 0.001), and persisted when examined for infectious and non-infectious disease diagnoses (5% and 11.3% higher in White patients, respectively). Minimal differences were found when stratified by SVI.

**Conclusion:**

Large differences in antibiotic utilization were observed by race but were not as pronounced by SVI. Additional factors such as age, diagnosis, and regional variability may need to be further examined in future analyses. Although these disparities were observed, our internal mortality data for infectious diseases shows no differences by race. Additional investigation is warranted to determine appropriateness of antibiotic use in each population to further elucidate on the disparities identified in this study.

**Disclosures:**

**All Authors**: No reported disclosures

